# Editorial: 3Rs—Strategies for reduction and refinement of animal studies

**DOI:** 10.3389/fphar.2023.1200965

**Published:** 2023-04-17

**Authors:** Eleonore Fröhlich, George D. Loizou

**Affiliations:** ^1^ Center for Medical Research, Medical University of Graz, Graz, Austria; ^2^ Health and Safety Executive, Bootle, United Kingdom

**Keywords:** replacement, reduction, refinement, physiologically-based kinetic (PBK) models, machine learning

Ethical guidelines in animal experimentation were defined as early as 1831 by the British psychologist Marshall Hall (Gorzalczany and Rodriguez Basso, 2021). The first concept of the 3Rs, which stands for replacement, reduction and refinement of animal studies, was presented in 1957 at the Universities Federal of Animal Welfare (UFAW) Symposium. An important step in the implementation of the concept in the member states was achieved by the adoption of the European Directive 2010/63/EU on the protection of animals used for scientific purposes in September 2010. It is a legal obligation, to apply the 3Rs in all aspects of the care and use of animals (https://ec.europa.eu/environment/chemicals/lab_animals/index_en.htm). Its content is similar to existing guidelines in numerous countries worldwide.

The 3Rs comprise a spectrum of very different strategies, ultimately resulting in the full replacement of animals in research and toxicological evaluation with human volunteer studies, tissues and established cell lines, and mathematical and computer models (Camenzind and Eggel, 2022). Partial replacement is achieved by using animals that, based on current scientific thinking, are not considered capable of experiencing suffering (e.g., bacteria, fungi, *drosophila*, nematode worms and embryonic and fetal forms of vertebrates). Reduction aims to achieve the best statistically significant result with the least number of animals by choosing an appropriate testing method and a properly designed study protocol. It also includes methods to maximize knowledge gain per animal used (e.g., longitudinal imaging, multi-omics technology). Refinement aims to minimize the amount of harm and suffering inflicted on research animals. This includes reduction in stress situations (e.g., improved handling of laboratory animal, environmental enrichment, entertainment in the enclosure with balls, hay, etc.), improved anesthesia and analgesia, and the setting of clear criteria for curtailing testing.

The contributions to this Research Topic focused on mathematical and computer models (physiologically based kinetic modeling (PBK), machine-learning algorithms and establishment of databases) to replace animal studies.

Widjaja et al. studied the kinetics of glutathione-dependent adduct formation of the pyrrolizidine alkaloid (PA) senecionine to determine the mechanism of hepatotoxicity of PA-N-oxides when reduced to their parent PAs. The parent PAs are bioactivated to pyrrole intermediates which form protein and DNA adducts. The rate of glutathione-dependent adduct formation of senecionine was determined in rat liver microsomal fractions and used as input into a PBK model. The authors reported that the decrease in glutathione-adduct formation increased the level of the toxic protein and DNA adducts. In a second PBK study, metabolism of the herbicide clethodim to the metabolite 3-chloroallyl alcohol (3-CAA) was investigated Conolly et al. The authors developed a PBK model using read-across information from a structurally related chemical and characterized the relationships between applied dose, target tissue dose, and maximum tolerated dose for studies intended to support regulatory evaluation of 3-CAA. Both studies illustrated how PBK modeling could help to replace animal studies.

The study by Liu et al. aimed to predict reproductive toxicity for risk assessment of environmental and industrial chemicals. Based on the datasets of 275 chemicals from the ToxRefDB (Toxicity Reference Database), predictive models using seven machine-learning algorithms (decision tree, decision forest, random forest, k-nearest neighbors, support vector machine, linear discriminant analysis, and logistic regression) were developed. These models demonstrated some predictive power for predicting potential activity of chemicals in rat multigeneration reproductive toxicity studies. The authors highlighted the importance of building a consensus model on the individual models and indicated the need for improvement due to the complicated mechanisms of reproductive toxicity.

Van Mulders et al. presented the Belgian project RE-Place, which is based on innovative technologies such as computer modeling, high throughput testing, omics, and sophisticated cell cultures, for the reduction or replacement of animal studies. National activities regarding the 3Rs initiated by the German Centre for the Protection of Laboratory Animals (“AnimAlt-ZEBET,” a database compiling methods linked to the 3Rs), and by Norway (“Norecopa” a platform to stimulate the 3Rs) the “RE-Place project” is the most recent database and launched in 2017 by the Flemish government. Organizations become members after registration and validation, whereupon members are granted access to an open access database, where structural information about a prospective method must be submitted. Methods are categorized as *in vitro*/*ex vivo*, *in silico*, *in vivo* using “lower” animals, in chemico and others. Model status should be specified as “in development,” “history of use,” “internally validated,” “published in peer-reviewed journals,” “submitted to external party” and “validated by external party.” Since May 2022, the database has received 157 *in vitro*/*ex vivo*, 15 *in silico*, 7 *in vivo* and 5 in chemico methods. The majority (123) are described in peer-reviewed journals, 64 internally validated, 60 have a history of use, 37 under development and 13 validated by an external party. The project is ongoing and continually optimized but the ultimate outcome will also greatly depend on future funding.

A PubMed search with the keywords (“3Rs” AND “*in vitro*”) OR (“3Rs” AND “*ex vivo*”) or “3Rs” AND “*in silico*” between 2011 (release of the Directive 2010/63/EU) and March 2023 resulted in 366 hits for the *in vitro*/*ex vivo* studies and 64 hits for *in silico* studies. The number of *in silico* methods reported in the RE-Place project database was even lower, indicating that these models are not as widely accepted as the biological studies. Therefore, the articles of this Research Topic represent an important gain in knowledge.
